# Effects of a Brief Web-Based “Social Norms”-Intervention on Alcohol, Tobacco and Cannabis Use Among German University Students: Results of a Cluster-Controlled Trial Conducted at Eight Universities

**DOI:** 10.3389/fpubh.2021.659875

**Published:** 2021-05-14

**Authors:** C. R. Pischke, S. M. Helmer, H. Pohlabeln, S. Muellmann, S. Schneider, R. Reintjes, A. Schmidt-Pokrzywniak, M. Girbig, A. Krämer, A. Icks, U. Walter, H. Zeeb

**Affiliations:** ^1^Institute of Medical Sociology, Centre for Health and Society, Medical Faculty, Heinrich Heine University Düsseldorf, Düsseldorf, Germany; ^2^Institute of Health and Nursing Science, Charité - Universitätsmedizin Berlin, Corporate Member of Freie Universität Berlin, Humboldt-Universität zu Berlin, and Berlin Institute of Health, Berlin, Germany; ^3^Department Prevention and Evaluation, Leibniz Institute for Prevention Research and Epidemiology - BIPS, Bremen, Germany; ^4^Mannheim Institute of Public Health, Social and Preventive Medicine, Ruprecht-Karls-University Heidelberg, Mannheim, Germany; ^5^Department of Health Sciences, Hamburg University of Applied Sciences, Hamburg, Germany; ^6^Martin-Luther-University Halle-Wittenberg, Halle, Germany; ^7^Institute and Policlinic of Occupational and Social Medicine, Technical University Dresden, Dresden, Germany; ^8^Department of Health Sciences, Bielefeld University, Bielefeld, Germany; ^9^Institute for Health Services Research and Health Economics, Centre for Health and Society, Medical Faculty, Heinrich Heine University Düsseldorf, Düsseldorf, Germany; ^10^Institute for Health Services Research and Health Economics, German Diabetes Center, Düsseldorf, Germany; ^11^Institute for Epidemiology, Social Medicine and Health Systems Research, Hannover Medical School, Hanover, Germany; ^12^Faculty 11 Human and Health Sciences, University of Bremen, Health Sciences, Bremen, Germany

**Keywords:** “social norms”-intervention, University context, cluster-controlled trial, substance use, alcohol, cannabis

## Abstract

**Background and Aim:** “Social norms” (SN)-interventions are aimed at changing existing misperceptions regarding peer substance use by providing feedback on actual norms, thereby affecting personal substance use. It is unknown whether SN-intervention effects previously demonstrated in US students can be replicated in German students. The aim of the INSIST-study was to examine the effects of a web-based SN-intervention on substance use.

**Design:** Cluster-controlled trial.

**Setting:** Eight Universities in Germany.

**Participants and Measurements:** Students were recruited at four intervention vs. four delayed intervention control Universities. 4,463 students completed baseline, 1,255 students (59% female) completed both baseline and 5-months follow-up web-based surveys on personal and perceived peer substance use. Intervention participants received feedback contrasting personal and perceived peer use with previously assessed use and perceptions of same-sex, same-university peers. Intervention effects were assessed *via* multivariable mixed logistic regression models.

**Findings:** Relative to controls, reception of SN-feedback was associated with higher odds for decreased alcohol use (OR: 1.91, 95% CI 1.42-2.56). This effect was most pronounced in students overestimating peer use at baseline and under or accurately estimating it at follow-up (OR: 6.28, 95% CI 2.00-19.8). The OR was 1.33 (95% CI 0.67-2.65) for decreased cannabis use in students at intervention Universities and was statistically significant at 1.70 (95% CI 1.13-2.55) when contrasting unchanged and decreased with increased use. Regarding tobacco use and episodes of drunkenness, no intervention effects were found.

**Conclusions:** This study was the first cluster-controlled trial suggesting beneficial effects of web-based SN-intervention on alcohol and cannabis use in a large sample of German University students.

**Clinical Trial Registration:** The trial registration number of the INSIST-study is DRKS00007635 at the “German Clinical Trials Register.”

## Introduction

It has previously been argued that the University setting is a high-risk environment for substance use due to the opportunity to use (1). Wicki and colleagues (2) reviewed the role of the University environment and student characteristics with regard to alcohol use at European campuses, including results of 65 studies. They found that alcohol was mainly consumed during social gatherings and that social motives for drinking were important. Alcohol and the use of other substances is perceived to be part of students' life and personally engaging in it is perceived to be adequate behavior to match the norm of peer behavior and to maintain conformity with the peer group (3). Harmful substance use behavior is prevalent among German University students. In a survey among students of 16 Universities in the German federal state of North Rhine-Westphalia, 80% reporting heavy drinking (4). Forty percent of the surveyed students had smoked (15% former, and 23% current smokers). Moreover, 41% of students had used cannabis at least once in their lifetime (5).

International research suggests that University students tend to overestimate both the perceived quantity (*descriptive norm*) (6–8) and perceived acceptability (*injunctive norm*) (9) of alcohol and other substances used by their peers. Inaccurate perceptions can cause the individual to increase personal use in an attempt to match the personal behavior to the perceived peer norm. Overestimations of peer licit (6–8) and illicit substance use (10–12) and associations with increased personal substance use among University students have been demonstrated in US and European studies (8, 10, 13).

Social norms (SN)-interventions involve personalized normative feedback (PNF) contrasting personal norms or perceptions of substance use and attitudes toward use among peers with data on actual use and attitudes in the peer group (14–16). Data on perceived attitudes and use, as well as actual attitudes and use, are assessed prior to the development of the feedback (15). PNF is then typically composed of three main components: (a) a student's self-reported own frequency of substance use, (b) a student's perceptions of substance use in the peer group, (c) actual frequencies of substance use in the peer group (17). Findings of SN-intervention studies suggest that participation in PNF leads to a reduction of social pressure on the individual and may consequently reduce personal substance use (18, 19). Recent studies have attempted to further unravel the effects of individual intervention components of SN-interventions and to identify optimal combinations of components. For example, one study examined the added benefit of an intervention combining descriptive-norms-feedback with injunctive-norms-feedback compared to a descriptive-norms-feedback only intervention. The study found that the combined intervention did not predict less drinking 2 weeks post-intervention compared to the single-component intervention (20). Another study investigated whether a full PNF compared to only providing the social comparison information to heavy drinkers was of similar effectiveness in reducing alcohol use. Results suggested that information regarding the discrepancy between actual drinking and the perceived drinking norm (i.e., “most students do not drink as much as you think they do”) did not have to be provided to reduce normative misperceptions (17).

There is already evidence regarding the effects of full PNF and various components and modalities of PNF on alcohol use in middle-aged adults (21) and students enrolled in the North-American college system (19). Specifically, the meta-analysis of 19 randomized controlled trials, including 8,095 adults, conducted by Riper et al. (21) revealed that individuals participating in internet-based interventions for adult problem drinking displayed a greater mean decrease in standard units of alcohol consumed per week at follow-up (compared to controls). Interventions based on PNF alone appeared to be less effective in promoting maintenance of low-risk drinking behavior compared to internet-based interventions based on therapeutic principles. Neighbors et al. (19) examined the efficacy of gender-specific vs. gender-nonspecific PNF in 818 heavy-drinking freshmen in a randomized controlled trial over the course of 2 years. They found that compared to controls, gender-specific biannual PNF was associated with reduced weekly drinking which was partially mediated by perceived norms that had changed over time.

Despite a growing body of evidence coming from EU-based studies examining the effects of SN-interventions, relatively little continues to be known about the effects of PNF in students enrolled at European Universities since the publication of the article of (3). Particularly in Germany, the effects of PNF on substance use continue to not be well-understood.

Furthermore, we assume that results of US studies on the effects of SN-interventions cannot simply be generalized to the European University environment. Comparisons between Germany and the US in the general population reveal differences between both countries in substance use prevalence which form the basis of normative feedback [Germany: larger percentage of respondents reporting current drinking and heavy drinking, US: respondents reported more alcohol-related problems at matched alcohol volume levels (22)]. A comparison between Sweden and the US suggests that, despite a higher alcohol use prevalence in Sweden, research from the US is generalizable to Swedish students and vice versa due to similar patterns between etiological predictors and outcomes (23). It is, however, doubtful whether this generalization can be extended to the German student population. One major difference that has an impact on the perception of use is certainly that, in Germany, only about 10% of German students live on campus and the majority of students live off-campus (24). Furthermore, the minimum legal drinking age differs between countries (21 vs. 18) and the regulations and policies at Universities vary between countries. Hence, social norms regarding substance use may be shaped less by substance use behavior visible in the proximal vicinity of students (i.e., on-campus) but probably more during off-campus activities (e.g., nightlife, private parties). Furthermore, the drinking occasions differ between countries. For instance, normative feedback interventions from the US focus on drinking at 21st birthday events known as dangerous drinking traditions that shape drinking behavior there (25), but the 21st birthday is not particularly celebrated in Germany.

A systematic review by Berman et al. (26) recently examined the effects of mobile interventions on risky drinking among University students (compared to controls) and included seven studies examining the effects of interventions employing varying modalities (text messages: *n* = 4, interactive voice response: *n* = 1, smartphone apps: *n* = 2). This review included one study conducted in Sweden by Andersson [(27), *n* = 1.678] examining the effects of different modalities of PNF (and protective behavioral strategies) on peak blood alcohol concentrations. Compared to controls, both the interactive voice response (IVR)- and the internet-based interventions led to a small but significant overall reduction in peak blood alcohol concentrations at the 6-week follow-up. A Swiss study investigated the long-term efficacy of an internet-based brief intervention, including normative and personalized feedback, for decreasing alcohol use among men assessing the number of drinks consumed per week and the occasions that men engaged in binge drinking (28). They found no differences between the intervention and control group regarding the number of drinks consumed per week and the prevalence of binge drinking at follow-up. The “Social Norms Intervention for the prevention of Polydrug usE” (SNIPE)-study was the first multi-national European study demonstrating the feasibility of this type of intervention on alcohol, tobacco, and illicit drugs in seven European countries (29) and demonstrating misperceptions regarding various substances [e.g., (10, 30)]. However, intervention effects were not evaluated in this study. Therefore, we conducted the INSIST (“INternet-based Social norms-Intervention for the prevention of substance use among Students”)-study to investigate intervention effects of the previously developed SN-intervention on misperceived social norms and the frequency of licit and illicit substance use among German University students enrolled at four intervention Universities compared to students enrolled at four delayed intervention control Universities (31).

The research questions were (a) whether students participating in the intervention reported lower rates of licit and illicit substance use (i.e., alcohol, tobacco, and cannabis consumption, episodes of drunkenness) at follow-up than those not participating in the intervention and (b) whether misperceptions of peer substance use were reduced as a consequence of participating in the intervention.

## Methods

### Participants and Procedures

Ethical approval was obtained from institutional review boards of all participating Universities. Data protection was monitored by the local data protection agency in the city state of Bremen. Eight Universities in four regions participated in the study (Hamburg University of Applied Sciences, Hannover Medical School, University of Bielefeld, Heinrich Heine University Duesseldorf, Martin-Luther-University Halle-Wittenberg, Technical University Dresden, Heidelberg University, Mannheim University). In each region, one University served as intervention, one as comparison site. Within a geographical area, intervention and control Universities were determined by random selection. Intervention and control Universities in each of the four regions were located in different cities (*intervention sites:* Hamburg, Bielefeld, Heidelberg, Halle vs. *control sites:* Hannover, Duesseldorf, Mannheim, Dresden). We had no consistent information on usual substance use prevalence at the included Universities, hence the only comparative data we have result from the current study.

**Figure 1:**
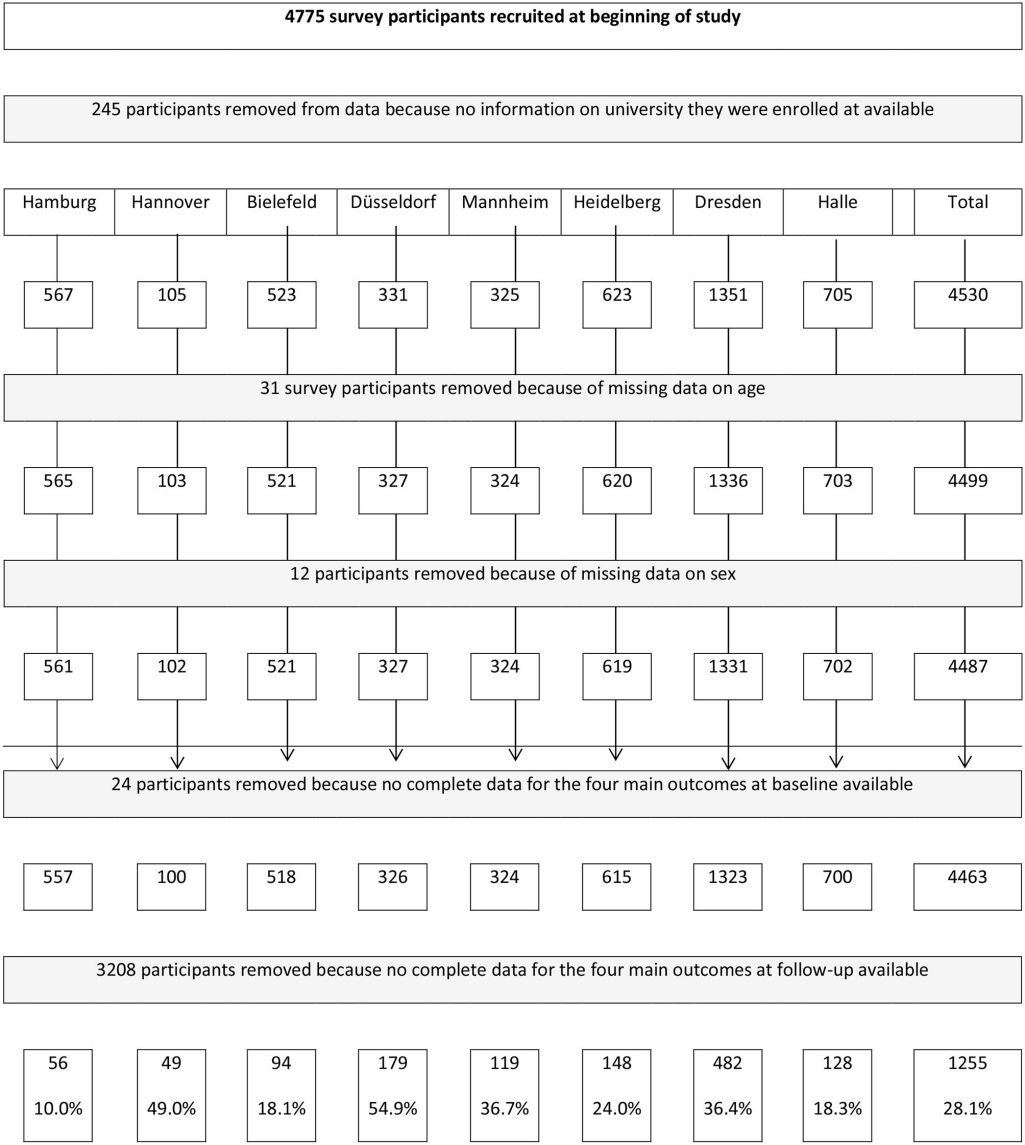
Flow chart of participants through the trial and analyses.

Recruitment for the study started in January 2014 (31). In the study, we had one overarching recruitment strategy across the participating Universities. At each University, one local student was part of the study staff and in charge of recruitment *via* email, the Universities' websites, intranet, or student e-learning platforms. Additional public recruitment channels included local newspaper articles, local radiobroadcasts, and student newsletters. Moreover, students were personally invited to participate in the study in seminars by University staff. Further, print-materials were used to recruit students as well as social media channels of Universities (i.e., Facebook). Students were included in the study if they were enrolled at one of the participating Universities, were aged 18 years and older, had access to the internet and an email-address. After registering onto the website, students received an email containing a hyperlink to the German language survey website where students could enter their email-address and choose their respective University and gender (female, male, or other). This information was then used to create the individualized University- and gender-specific SN-feedback which was delivered during the intervention. Students were also told that they could withdraw from the study at any time. Overall, 167,686 students were enrolled at the participating Universities. 7,088 students (4%) registered on the study website, and 4,463 completed the baseline survey (2.7% out of all enrolled students) (see Figure 1 and Table 1).

**Table 1:** Sample characteristics by group and gender at baseline.

	**Males**		**Females**		**All**	
	**CG (*n* = 372)**	**IG (*n* = 140)**	**CG (*n* = 457)**	**IG (*n* = 286)**	**CG (*n* = 829)**	**IG (*n* = 426)**
	***N* (%)**	***N* (%)**	***N* (%)**	***N* (%)**	***N* (%)**	***N* (%)**
**Age**						
≤20 years	77 (20.7)	26 (18.6)	105 (23.0)	59 (20.6)	182 (22.0)	85 (20.0)
21-25 years	206 (55.4)	81 (57.9)	250 (54.7)	157 (54.9)	456 (55.0)	238 (55.9)
26-30 years	69 (18.5)	23 (16.4)	82 (17.9)	57 (19.9)	151 (18.2)	80 (18.8)
≥31+ years	20 (5.4)	10 (7.1)	20 (4.4)	13 (4.5)	40 (4.8)	23 (5.4)
**Field of study**						
Arts	38 (10.2)	16 (11.4)	73 (16.0)	59 (20.6)	111 (13.4)	75 (17.6)
Business and Law	51 (13.7)	13 (9.3)	37 (8.1)	17 (5.9)	88 (10.6)	30 (7.0)
Engineering	87 (23.4)	10 (7.1)	44 (9.6)	10 (3.5)	131 (15.8)	20 (4.7)
Medicine/Health	52 (14.0)	18 (12.9)	79 (17.3)	68 (23.8)	131 (15.8)	86 (20.2)
Natural Science	57 (15.3)	39 (27.9)	70 (15.3)	47 (16.4)	127 (15.3)	86 (20.2)
Social Sciences	54 (14.5)	23 (16.4)	122 (26.7)	67 (23.4)	176 (21.2)	90 (21.1)
Maths/Informatics	30 (8.1)	16 (11.4)	16 (3.5)	12 (4.2)	46 (5.5)	28 (6.6)
Others	3 (0.8)	5 (3.6)	16 (3.5)	6 (2.1)	19 (2.3)	11 (2.6)
**University**						
Hamburg		19 (13.6)		37 (12.9)		56 (13.1)
Bielefeld		28 (20.0)		66 (23.1)		94 (22.1)
Heidelberg		61 (43.6)		87 (30.4)		148 (34.7)
Halle		32 (22.9)		96 (33.6)		128 (30.0)
Hannover	18 (4.8)		31 (6.8)		49 (5.9)	
Düsseldorf	72 (19.4)		107 (23.4)		179 (21.6)	
Mannheim	58 (15.6)		61 (13.3)		119 (14.4)	
Dresden	224 (60.2)		258 (56.5)		482 (58.1)	
**Residence**						
Living with other students	135 (36.3)	40 (28.6)	129 (28.2)	76 (26.6)	264 (31.8)	116 (27.2)

*CG, control group; IG, intervention group*.

### Web-Based Baseline and Follow-Up Surveys

In the web-based questionnaire, students were asked to answer questions regarding their personal and perceived gender-specific substance use of peers at their University toward using the following substances: Alcoholic beverages, tobacco products, waterpipe, cannabis, non-prescribed medications to improve academic performance, non-prescribed sedatives or sleeping pills, synthetic cannabis, cocaine, ecstasy, other amphetamine-type stimulants, hallucinogens, and inhalants. Furthermore, two types of polydrug use were assessed (i.e., simultaneous use of alcohol and tobacco, of alcohol and illicit substances, such as cannabis, ecstasy, or cocaine). Furthermore, students were asked how often in the last 2 months they drank until they felt drunk. The choice of substances included was based on the Alcohol, Smoking, and Substance Involvement Screening Test (ASSIST), developed by the World Health Organization (32) and was slightly adjusted as described in a previous trial (31). Licit and illicit substances were described with a list of examples and, if applicable, trade or street names for each substance. Along with the items on alcohol use, participants were provided with a definition of an alcoholic drink as 0.33 L beer, a small bottle of a ready to drink beverage (0.275L), a small cocktail (0.2L, containing 4cl alcohol), a glass of wine/sparkling wine (0.125L), and a shot of spirits (0.4L).

Referring to this range of substances, students were asked to report their personal substance use. Response options ranged for alcohol (frequency and drank until felt drunk), tobacco, waterpipe, cannabis and polydrug use from “never used in their lives,” “used but not in the last 2 months,” “used once or twice in the last 2 months,” “used every 2 weeks in the last 2 months,” “used once or twice in the last week in the last 2 months,” “3-4 times per week in the last 2 months,” and “daily or almost daily in the last 2 months.” The following categories were used for non-prescribed medications and sedatives, synthetic cannabis, cocaine, ecstasy, other amphetamines, hallucinogenic drugs, inhalants: “never used in their lives,” “have used but not in the last 2 months,” “have used 1-3 times in the last 2 months,” “have used weekly or more often in the last 2 months,” “have used daily or almost every day in the last 2 months.” Furthermore, students were asked to indicate their perceptions of gender-specific behaviors (*descriptive norm*, the perception of quantity and frequency of substance use in the peer group) among their peers regarding the frequency of alcohol, tobacco, waterpipe, and illicit substance use. To assess the *descriptive norm*, students were asked to imagine all students of their University (100% of the same gender as the respondent) and to estimate the frequency of use of various substances during the last 2 months in their peer group. They were asked to distribute the percentages of students to the same categories for reporting personal use. The questions and the used reference groups followed the same principle of previous SN surveys (29, 33). However, the response modalities differed substantially based upon discussions with the project-own advisory board consisting of international SN researchers (31).

All questions referred to a time period of 2 months prior to assessment. The time frame of the previous 2 months was used as this covered the period when students attended University, as planned in the schedule of data collection. The follow-up survey took place 5 months post-baseline employing the same items. Students at intervention Universities were asked one additional item assessing whether they remembered the content of the normative feedback (including an example). For this article, despite having collected data on prevalences of all the substances listed above, pre-/post-comparisons were only calculated for the three main substances alcohol, tobacco, and cannabis because the prevalence for most of the other substances was too low for comparing use before and after the intervention.

### Web-Based SN-Intervention

The intervention developed during the earlier SNIPE-study was further adapted to better fit the German University context based on a focus group discussion with seven students from two Universities in Northern Germany (31). Based on the baseline data on descriptive and injunctive norms related to substance use at the respective University, a gender-specific, normative feedback was developed and sent to students enrolled at the four intervention Universities 8 weeks after completion of the baseline survey. Five months post-baseline (August 2014), students at the delayed intervention control Universities were given access to this feedback. The feedback website consisted of several different main pages that were accessible *via* a navigation menu. Each main page contained information about a different substance (i.e., alcohol, tobacco, cannabis) and was divided into a personalized feedback and a gender- and University-specific feedback. The personalized feedback included the individual information regarding own substance use and the perception of use in the peer group (of the same gender and University) reported by students. If students did not fill out these questions in the baseline questionnaire beforehand, they were informed that an individual feedback could not be given. The gender- and University-specific feedback visualized the perceived peer substance use (of the majority of students of the same gender, same University) estimated by the student. This information was contrasted with the actual substance use pattern of students of the same gender and same University as assessed in the baseline questionnaire. These two comparisons formed the descriptive norms feedback. Furthermore, students received information about the injunctive norms of same-gender peers at their Universities.

### Statistical Analysis

Descriptive analysis was performed using tabulations for personal alcohol, tobacco and cannabis use and episodes of drunkenness. Furthermore, we calculated the percentages of respondents who (a) underestimated/accurately estimated peer use both at baseline and at follow-up, (b) overestimated peer use at baseline and underestimated or accurately estimated use at follow-up, (c) underestimated/accurately estimated peer use at baseline and overestimated use at follow-up and, (d) overestimated peer use at both time points regarding alcohol, tobacco, and cannabis. For this, the gender-specific substance use prevalence at the respective University was contrasted with the perception of use of the majority of students of the same gender at the same University. If a student reported “other” gender, the data of all students were used for comparison.

To evaluate intervention efficacy, substance use pre- and post-intervention among students at intervention Universities was compared to the use reported by students enrolled at control Universities. Our main focus was to assess whether consumption of alcohol, tobacco, cannabis and episodes of drunkenness had increased, not changed or decreased from baseline (T0) to follow-up (T1), contrasting intervention and control Universities. Hence, the effect of the intervention on the main outcome (consumption decreased) was assessed by means of multivariable mixed logistic regression models (corrected for clustering at the University level), considering age, gender, as well as baseline substance use, as covariates. Subgroup analyses were conducted to analyse differences in changes in alcohol, cannabis, and tobacco use by gender and by changes in perceptions of peer use over the follow-up. These analyses were stratified by the four groups of peer-use perception combinations. Furthermore, following a similar approach, we used the less stringent criterion “consumption did not increase” (i.e., unchanged or decreased). Odds ratios and 95% confidence intervals were estimated from the models. SAS statistical software (34) was used for all quantitative analyses.

## Results

A summary of the sample characteristics can be found in Table 1 and further details on distinct consumption patterns of concurrent and non-concurrent substance use analyzed with cluster analysis can be found in the article by Schilling et al. (35). A total of six homogeneous groups were identified: “Alcohol Abstainers” (10.8%), “Drinkers Only” (48.2%), “Drinkers and Cigarette Smokers” (14.6%), “Cannabis and Licit Substance Users” (11.2%), “Hookah Users with Co-Use” (9.8%) and “Illicit Substance Users with Co-Use” (5.4%) (35). For this article, the analytic population comprised only students with complete baseline and follow-up information. Intervention participants in the analytic sample (*n* = 426; HAW Hamburg: *n* = 56, University Bielefeld: *n* = 94, University Heidelberg: *n* = 148, MLU Halle: *n* = 128, also see Table 1) received feedback contrasting personal and perceived peer use with previously assessed use and perceptions of same-sex, same-University peers. Of those receiving feedback, over one third reported not remembering receiving a normative feedback (HAW Hamburg: 43.1%, University Bielefeld: 34.3%, University Heidelberg: 33.6%, MLU Halle: 27.3%) (not shown). Eight-hundred and twenty-nine participants at the control Universities completed the follow-up survey with most students recruited in Dresden (*n* = 482).

1,255 students (59% female) completed both baseline and follow-up web surveys. At baseline, about 75% were under the age of 25 years. Slightly fewer students at intervention Universities (27.2%) than at control Universities (31.2%) reported living with other students with marked differences between individual sites. The field of study (assessed at baseline) varied broadly, as some Universities were medical schools, others had a strong focus on social sciences or engineering (for further detail, see Table 1).

Prevalences for substance use at baseline by group and gender can be found in Table 2. Alcohol and tobacco were the substances most commonly used, with markedly higher prevalences among male students and a slightly worse overall profile for control Universities. Use of performance-enhancing drugs, sedatives, and synthetic cannabis was reported very rarely.

**Table 2:** Baseline prevalence of licit and illicit substance use by group and gender.

	**Males**		**Females**		**All**	
	**CG (*n* = 372)**	**IG (*n* = 140)**	**CG (*n* = 457)**	**IG (*n* = 286)**	**CG (829)**	**IG (*n* = 426)**
	***N* (%)**	***N* (%)**	***N* (%)**	***N* (%)**	***N* (%)**	***N* (%)**
**Alcohol use**						
3x/week or more	98 (26.3)	28 (20.0)	55 (12.0)	30 (10.5)	153 (18.5)	58 (13.6)
**Tobacco use**						
3x/week or more	49 (13.2)	15 (10.7)	52 (11.4)	31 (10.8)	101 (12.2)	46 (10.8)
**Cannabis use**						
At least 1x/week	27 (7.3)	8 (5.7)	12 (2.6)	6 (2.1)	39 (4.7)	14 (3.3)
**Episodes of drunkenness**						
At least 1x/week	58 (15.6)	19 (13.6)	34 (7.4)	20 (7.0)	92 (11.1)	39 (9.2)
**Academic performance enhancing drugs**						
Use in the past 2 months	4 (1.1)	0 (0)	4 (0.9)	1 (0.3)	8 (1.0)	1 (0.2)
**Sedatives/sleeping pills**						
Use in the past 2 months	2 (0.5)	1 (0.7)	8 (1.8)	5 (1.7)	10 (1.2)	6 (1.4)
**Synthetic cannabis**						
Use in the past 2 months	3 (0.8)	1 (0.7)	2 (0.4)	2 (0.7)	5 (0.6)	3 (0.7)

*CG, Control group; IG, Intervention group*.

With regard to changes in perceptions of peer use, there was a clear pattern of overestimated peer use, both, at baseline and at follow-up, and across all substances. For example, 303 (71.1%) respondents at intervention and 651 (78.5%) respondents at control Universities overestimated alcohol use at both time points. Overall, only 29 persons accurately or under-estimated peer alcohol use at both time points. Only for cannabis use, the picture was somewhat different with 19.1% of controls and 17.4% of intervention participants accurately or under-estimating peer use at both time points (Table 3).

**Table 3:** Change of alcohol, tobacco, cannabis consumption and change in episodes of drunkenness (increased, unchanged, decreased) 5 months post-intervention (stratified by estimation of peer use at T0/T1).

	**Stratified by estimation of peer use at baseline (T0) and follow-up (T1)**									
	**Total analysis group**		**Under-/accurately at T0**		**Overestimated at T0**		**Under-/accurately at T0**		**Overestimated at T0**	
	**(*****n*** **= 1,255)**		**Under-/accurately at T1**		**Under-/accurately at T1**		**Overestimated at T0**		**Overestimated at T1**	
	**CG**	**IG**	**CG**	**IG**	**CG**	**IG**	**CG**	**IG**	**CG**	**IG**
	***N* (%)**	***N* (%)**	***N* (%)**	***N* (%)**	***N* (%)**	***N* (%)**	***N* (%)**	***N* (%)**	***N* (%)**	***N* (%)**
**Alcohol consumption**										
Increased	211 (25.5)	97 (22.8)	6 (37.5)	4 (30.8)	7 (15.2)	6 (13.3)	17 (42.5)	3 (17.6)	160 (24.6)	77 (25.4)
Unchanged	466 (56.2)	209 (49.1)	5 (31.3)	6 (46.2)	32 (69.6)	18 (40.0)	14 (35.0)	8 (47.1)	374 (57.5)	147 (48.5)
Decreased	152 (18.3)	120 (28.2)	5 (31.3)	3 (23.1)	7 (15.2)	21 (46.7)	9 (22.5)	6 (35.3)	117 (18.0)	79 (26.1)
Total	829 (100)	426 (100)	16 (100)	13 (100)	46 (100)	45 (100)	40 (100)	17 (100)	651 (100)	303 (100)
**Tobacco consumption**										
Increased	139 (16.8)	54 (12.7)	5 (12.8)	2 (9.5)	4 (8.0)	3 (7.5)	20 (17.4)	4 (12.5)	93 (17.4)	39 (13.9)
Unchanged	598 (72.1)	338 (79.3)	28 (71.8)	18 (85.7)	39 (78.0)	34 (85.0)	83 (72.2)	26 (81.3)	381 (71.2)	216 (77.1)
Decreased	92 (11.1)	34 (8.0)	6 (15.4)	1 (4.8)	7 (14.0)	3 (7.5)	12 (10.4)	2 (6.3)	61 (11.4)	25 (8.9)
Total	829 (100)	426 (100)	39 (100)	21 (100)	50 (100)	40 (100)	115 (100)	32 (100)	535 (100)	280 (100)
**Cannabis consumption**										
Increased	113 (13.6)	34 (8.0)	23 (14.5)	2 (2.7)	13 (17.6)	6 (11.8)	12 (14.8)	1 (5.0)	50 (12.2)	16 (7.3)
Unchanged	647 (78.0)	353 (82.9)	118 (74.2)	66 (89.2)	59 (79.7)	38 (74.5)	66 (81.5)	17 (85.0)	325 (79.3)	183 (83.6)
Decreased	69 (8.3)	39 (9.2)	18 (11.3)	6 (8.1)	2 (2.7)	7 (13.7)	3 (3.7)	2 (10.0)	35 (8.5)	20 (9.1)
Total	829 (100)	426 (100)	159 (100)	74 (100)	74 (100)	51 (100)	81 (100)	20 (100)	535 (100)	280 (100)
Episodes of drunkenness^*^										
Increased	147 (17.7)	62 (14.6)	4 (25.0)	1 (7.7)	4 (8.7)	6 (13.3)	15 (37.5)	3 (17.6)	112 (17.2)	44 (14.5)
Unchanged	472 (59.6)	243 (57.0)	9 (56.3)	9 (69.2)	31 (67.4)	23 (51.1)	17 (42.5)	9 (52.9)	366 (56.2)	175 (57.8)
Decreased	210 (25.3)	121 (28.4)	3 (18.8)	3 (23.1)	11 (23.9)	16 (35.6)	8 (20.0)	5 (29.4)	173 (26.6)	84 (27.7)
Total	829 (100)	426 (100)	16 (100)	13 (100)	46 (100)	45 (100)	40 (100)	17 (100)	651 (100)	303 (100)

*CG, Control group; IG, Intervention group*.

* *Stratified by estimation of frequency of alcohol use*.

### Intervention Effects

Regarding alcohol consumption, there were slight differences between the intervention and control groups, as 25.5% in the control group, but 22.8% of the intervention group reported increases from T0 to T1, and 18.3 vs. 28.2% reported decreased consumption (Table 3). Regarding decreased consumption, the OR was 1.91 (95% CI 1.42-2.56), and in the small group of students overestimating peer use at baseline and under or accurately estimating peer use at follow-up, the OR was 6.28 (95% CI 2.00-19.8) (Table 4). Non-significant findings were obtained when comparing the outcome “alcohol consumption not increased” between intervention and control groups (Table 5).

**Table 4:** Results of multivariable logistic regression models (total and stratified); Outcomes: alcohol, tobacco, cannabis consumption, and episodes of drunkenness *decreased* 5 months post-intervention.

	**Stratified by estimation of peer use at baseline (T0) and follow-up (T1)**									
	**Total analysis group**		**Under-/accurately at T0**		**Overestimated at T0**		**Under-/accurately at T0**		**Overestimated at T0**	
	**(*****n*** **= 1,255)**		**Under-/accurately at T1**		**Under-/accurately at T1**		**Overestimated at T0**		**Overestimated at T1**	
	**OR (95% CI)**	***P***	**OR (95% CI)**	***P***	**OR (95% CI)**	***P***	**OR (95% CI)**	**p**	**OR (95% CI)**	***P***
**Alcohol consumption**										
Intervention (IG vs. CG)	**1.91 (1.42-2.56)**	**<0.001**	^*^	^*^	**6.28 (2.00-19.8)**	**0.002**	1.82 (0.48-6.90)	0.371	1.86 (1.32-2.63)	<0.001
**Tobacco consumption**										
Intervention (IG vs. CG)	0.68 (0.38-1.22)	0.194	0.11 (0.00-2.10)	0.139	0.42 (0.03-6.00)	0.520	0.39 (0.06-2.47)	0.315	0.71 (0.31-1.60)	0.406
**Cannabis consumption**										
Intervention (IG vs. CG)	1.33 (0.67-2.65)	0.414	0.83 (0.26-2.65)	0.753	**11.7 (1.24-110)**	**0.032**	^*^	^*^	1.74 (0.44-6.95)	0.430
**Episodes of drunkenness**										
Intervention (IG vs. CG)	1.32 (0.98-1.80)	0.072	1.34 (0.00-253.0)	0.753	2.45 (0.63-9.48)	0.191	1.84 (0.41-8.26)	0.419	1.27 (0.89-1.82)	0.187

* *Sample size in this subgroup was too small to derive reliable estimates (algorithm did not converge)*.

*CG, Control group; CI, Confidence Interval; IG, Intervention group; OR, Odds ratio; p-value (derived by means of a multivariable logistic regression model, additionally adjusted for alcohol, tobacco, or cannabis consumption or episodes of drunkenness at baseline; University treated as random effect). Bold values indicate significant effects*.

**Table 5:** Results of multivariable logistic regression models (total and stratified); Outcome: consumption *not increased* 5 months post-intervention.

	**Stratified by estimation of peer use at baseline (T0) and follow-up (T1)**									
	**Total analysis group**		**Under-/accurately at T0**		**Overestimated at T0**		**Under-/accurately at T0**		**Overestimated at T0**	
	**(*****n*** **= 1,255)**		**Under-/accurately at T1**		**Under-/accurately at T1**		**Overestimated at T0**		**Overestimated at T1**	
	**OR (95% CI)**	***P***	**OR (95% CI)**	***P***	**OR (95% CI)**	***P***	**OR (95% CI)**	***P***	**OR (95% CI)**	***P***
**Alcohol consumption**										
Intervention (IG vs. CG)	1.36 (0.90-2.04)	0.147	1.22 (0.05-28.3)	0.896	1.20 (0.33-4.31)	0.776	3.23 (0.72-14.5)	0.371	1.14 (0.70-1.85)	0.52
**Tobacco consumption**										
Intervention (IG vs. CG)	1.34 (0.90-2.01)	0.152	1.46 (0.24-8.97)	0.680	1.84 (0.18-18.7)	0.603	1.69 (0.52-5.57)	0.384	1.27 (0.82-1.98)	0.285
**Cannabis consumption**										
Intervention (IG vs. CG)	**1.70 (1.13**-**2.55)**	**0.011**	5.55 (1.25-24.6)	0.024	1.82 (0.51-6.51)	0.355	3.71 (0.43-32.2)	0.231	1.58 (0.86-2.89)	0.142
**Episodes of drunkenness**										
Intervention (IG vs. CG)	1.26 (0.91-1.75)	0.169	^*^	^*^	0.43 (0.09-2.00)	0.278	5.17 (0.71-37.6)	0.103	1.23 (0.83-1.80)	0.301

* *Sample size in this subgroup was too small to derive reliable estimates (algorithm did not converge)*.

*CG, Control group; CI, Confidence Interval; IG, Intervention group; OR, Odds ratio; p-value (derived by means of a multivariable logistic regression model, additionally adjusted for alcohol, tobacco, or cannabis consumption or episodes of drunkenness at baseline; University treated as random effect). Bold values indicate significant effects*.

For tobacco use, there were less obvious changes, with 72.1% in the control and 79.3% in the intervention group reporting no change in consumption (Table 3). The OR for decreased tobacco consumption between T0 and T1 was 0.68 (95% CI 0.38-1.22) and was not reduced across all categories of peer-use perception (Table 4). Combining the unchanged and decreased group into the “not increased” group led to non-significantly elevated OR favoring the intervention across all peer-use perception groups.

13.6% in the control group against 8.0% in the intervention group reported increased cannabis use at follow up, while the unchanged or decreased groups were of very similar size (Table 3). The OR was 1.33 (95% CI 0.67-2.65) for decreased consumption in the intervention group (Table 4), and was statistically significant at 1.70 (95% CI 1.13-2.55) when contrasting “not increased” vs. “increased” (Table 5). Here, the highest OR (11.7) were found in the group that under- or accurately estimated peer use at follow-up and had previously overestimated it (95% CI 1.24-110) (Table 4).

Finally, episodes of drunkenness were assessed and only small differences between intervention and control group participants were detected (Table 3) with an elevated OR of 1.32 for a decrease in the intervention against the control group (95% CI 0.98-1.80) and with some variation across peer-use perception groups (Table 4).

Comparing male and female students, the largest decreases were seen regarding episodes of drunkenness in both sexes and across groups. Changes of similar size were also seen for alcohol consumption, where women in the control group reported markedly higher decreases compared to men in the control group. Interestingly, the proportion of respondents indicating increase of alcohol consumption was also higher in females than in males in the control group. Cannabis consumption changed less among women than men, with about 80% of women and 75% of men reporting unchanged consumption at T1.

To assess sex-specific intervention effects, stratified models were calculated. We saw a significantly elevated odds ratio for decreased alcohol consumption at T1 among both men (OR 2.34) and women (OR 1.71). Most OR estimates were close to the null effect; only for cannabis an elevated OR of 2.02 (95% CI 1.13-3.59) among women was seen for the outcome of non-increased consumption while the result for men was unremarkable. Effects by gender are displayed in further detail in Tables 4.1, 5.1. However, the broadly overlapping confidence intervals do not allow any substantial interpretations about differences in risk estimates between male and female students.

**Table 4.1:** Gender-specific results of multivariable logistic regression models; Outcomes: alcohol, tobacco, cannabis consumption, and episodes of drunkenness decreased 5 months post-intervention.

	**Males**		**Females**	
	**OR (95% CI)**	***P***	**OR (95% CI)**	***P***
**Alcohol consumption**				
Intervention (IG vs. CG)	2.34 (1.44-3.80)	**<0.001**	**1.71 (1.18-2.47)**	**0.004**
**Tobacco consumption**				
Intervention (IG vs. CG)	0.82 (0.41-1.65)	0.581	0.58 (0.25-1.35)	0.206
**Cannabis consumption**				
Intervention (IG vs. CG)	1.64 (0.82-3.31)	0.164	1.07 (0.39-3.00)	0.893
**Episodes of drunkenness**				
Intervention (IG vs. CG)	1.33 (0.77-2.32)	0.310	1.38 (0.93-2.06)	0.110

*CG, Control group; CI, Confidence Interval; IG, Intervention group; OR, Odds ratio; p-value (derived by means of a multivariable logistic regression model, adjusted for age and alcohol, tobacco, or cannabis consumption or episodes of drunkenness at baseline; University treated as random effect). Bold values indicate significant effects*.

**Table 5.1:** Gender-specific results of the multivariable logistic regression models; Outcomes: alcohol, tobacco, cannabis consumption, and episodes of drunkenness *not increased* 5 months post-intervention.

	**Males**		**Females**	
	**OR (95% CI)**	***P***	**OR (95% CI)**	***P***
**Alcohol consumption**				
Intervention (IG vs. CG)	1.14 (0.68-1.91)	0.620	1.40 (0.83-2.36)	0.206
**Tobacco consumption**				
Intervention (IG vs. CG)	1.43 (0.79-2.61)	0.241	1.36 (0.89-2.10)	0.158
**Cannabis consumption**				
Intervention (IG vs. CG)	1.39 (0.78-2.49)	0.264	**2.02 (1.13**-**3.59)**	**0.017**
**Episodes of drunkenness**				
Intervention (IG vs. CG)	1.01 (0.56-1.83)	0.973	1.51 (0.98-2.31)	0.060

*CG, Control group; CI, Confidence Interval; IG, Intervention group; OR, Odds ratio; p-value (derived by means of a multivariable logistic regression model, adjusted for age and alcohol, tobacco, or cannabis consumption or episodes of drunkenness at baseline; University treated as random effect). Bold values indicate significant effects*.

As can be seen in the table for individuals overestimating use at T0 and under-/accurately estimating use at T1 (Table 6), there were only four participants with frequent use at T0 (three times per week or more) in the control group, of those two participants reduced their consumption by one category at T1 (50%). In the intervention group, three participants were in the highest frequency of use group, of those two reduced their frequency of use by one category at T1 (66.6%). Looking at participants with moderate frequency of use at T0 (2-8 times/month), of 24 participants in the control group, approximately one third remained in the same category, five participants (20.8%) reduced alcohol use. In the intervention group, a higher percentage of participants moved to the lower category at T1 (12 of 23, 52.2%). Looking at changes in frequency of use among individuals overestimating peer use at both time points, we can see in Table 7 that, in the control group, 141 participants with frequent use reduced their alcohol use (37 + 1, 26.9%) and 13 + 5 (40.9%) in the intervention group. Among individuals with moderate use, 8.5% reduced alcohol use and approximately twice as many in the intervention group (17.4%).

**Table 6:** Frequency of alcohol consumption at baseline and follow-up among individuals overestimating peer use at baseline and correctly or underestimating use at follow-up (CG: *n* = 46, IG: *n* = 45).

**Frequency of alcohol consumption**	**CG**								**IG**							
** 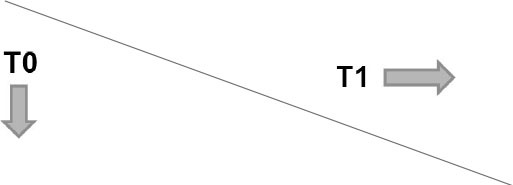 **	**At most**		**2-8x/**		**3x/week**		**All**		**At most**		**2-8x/**		**3x/week**		**All**	
	**1x/month**		**month**		**or more**				**1x/month**		**month**		**or more**			
	***N***	**%**	***N***	**%**	***N***	**%**	***N***	**%**	***N***	**%**	***N***	**%**	***N***	**%**	**N**	**%**
At most 1x/month	17	94.4	1	5.6	0	0	18	100.0	16	84.2	3	15.8	0	0	19	100.0
2-8x/month	5	20.8	16	66.7	3	12.5	24	100.0	12	52.2	11	47.8	0	0	23	100.0
3x/week or more	0	0	2	50.0	2	50.0	4	100.0	0	0	2	66.7	1	33.3	3	100.0
Total	22	47.8	19	41.3	5	10.9	46	100.0	28	62.2	16	35.6	1	2.2	45	100.0

*CG, Control group; CI, Confidence Interval; IG, Intervention group*.

**Table 7:** Frequency of alcohol consumption at baseline and follow-up among individuals overestimating peer use at baseline and follow-up (CG: *n* = 651, IG: *n* = 303).

**Frequency of alcohol consumption**	**CG**								**IG**							
** 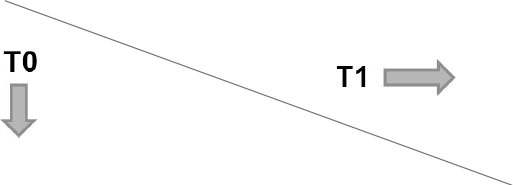 **	**At most**		**2-8x/**		**3x/week**		**All**		**At most**		**2-8x/**		**3x/week**		**All**	
	**1x/month**		**month**		**or more**				**1x/month**		**month**		**or more**			
	***N***	**%**	***N***	**%**	***N***	**%**	***N***	**%**	***N***	**%**	***N***	**%**	***N***	**%**	**N**	**%**
At most 1x/month	132	78.1	37	21.9	0	0	169	100.0	82	83.7	16	16.3	0	0	98	100.0
2-8x/month	29	8.5	256	75.1	56	16.4	341	100.0	28	17.4	107	66.5	26	16.1	161	100.0
3x/week or more	1	0.7	37	26.2	103	73.0	141	100.0	5	11.4	13	29.5	26	59.1	44	100.0
Total	162	24.9	330	50.7	159	24.4	651	100.0	115	38.0	136	44.9	52	17.2	303	100.0

*CG, Control group; CI, Confidence Interval; IG, Intervention group*.

## Discussion

Participation in a web-based PNF was associated with higher odds for decreased alcohol and cannabis use among students enrolled at intervention compared to delayed intervention control Universities. The observed intervention effect may be linked to the fact that, similar to North American and other European student populations (10, 13, 30, 36), German students at the eight Universities enrolled in this study misperceived alcohol and cannabis use in their peer group. As expected, the majority of students at all participating Universities perceived the use of both substances to be higher than the actually assessed prevalences at the Universities at both time points. Further, our results suggest that in the group that had overestimated alcohol use at baseline and under or accurately estimated peer alcohol use at follow-up, reductions in alcohol use were most pronounced.

In addition, an overall intervention effect could be detected for cannabis use among students at intervention compared to control Universities, especially when contrasting increased with decreased use over the course of the follow-up. Similar to the results for alcohol use, students in the group that moved to more accurate perceptions of peer cannabis use over the follow-up benefited the most from the intervention and decreased personal use. However, the numbers of students in the different categories for the analysis of the subgroups were very small. Our results should therefore be interpreted with caution and need to be replicated in a larger sample of German University students. Regarding tobacco use, a different picture emerged. Our study did not demonstrate an intervention effect pertaining to all the substances assessed and no variations by category of peer-use perception were observed. A combination of PNG with other behavior change strategies as part of multicomponent interventions may be more promising for influencing a wider range of substances used. However, it is difficult to separate treatment effects of intervention components and compare multi-component interventions to standalone approaches (37). Further, standalone online-interventions might not be effective enough to change long established addictive behaviors, such as tobacco use. Previous research suggests that interventions combining an online intervention with personal counseling led to higher satisfaction (38). It also remains unclear whether the INSIST-intervention challenged the misperceptions. There is a need to also add an assessment of pre- to post-changes in perceived norms to SN studies (15).

Hence, feedback provided to students in our study led to more accurate perceptions of peer alcohol and cannabis use in a relatively small group of students. These changed perceptions, in turn, appeared to be associated with reduced alcohol and cannabis use at 5-months follow-up. Similarly, a study by Su et al. (39) examined the effects of a campus-wide social marketing campaign on alcohol use among 4,172 college students and found that reading campaign messages was associated with more accurate perceptions of peer alcohol use. In addition, and probably, as a result of exposure to the campaign, students reported consuming fewer drinks per sitting and fewer blackouts due to binge drinking at 6-months follow-up. A controlled intervention study targeting Canadian University students found that changes in norm misperceptions at 3-months mediated the effect of e-CHECKUP TO GO, an intervention containing SN feedback and self-monitoring of drinking behavior, on drinking outcomes at 5-months follow-up (40). In a Swedish study, obtaining personalized normative feedback online and *via* IVR was associated with a significant reduction in peak blood alcohol concentrations after 6 weeks in University students (27), while a Swiss study did not find any effects on long-term alcohol use and binge drinking in young men between the ages of 20 and 25 years (28). A systematic review by Riper et al. (21) revealed that web-based interventions that were solely based on personalized normative feedback were less likely to be effective for adult problem drinkers than intervention strategies based on integrated therapeutic principles. Therefore, short web-based interventions aimed at changing misperceived norms may not be sufficient for people already involved in high-risk use (21). Thus, additional research is needed so that interventions can be optimized toward specific target groups. Further, we are not aware of comparable studies examining the effects of PNF on cannabis or tobacco use among University students. Because alcohol, tobacco, and cannabis are different substances associated with varying consequences at the individual level, as well as varying levels of public acceptance, PNF may not work in a unified way. For example, compared to the US, tobacco is less regulated and use is still more socially accepted in Germany, whereas cannabis is still an illegal drug and acceptance varies in different population groups in Germany. Acceptance of cannabis use appears to be higher in young adults aged 18-25, where almost 50% have used cannabis during their lifetime, while in the age group of 12-17 year-olds, only 10% have done so. Alcohol consumption is widely socially accepted with almost 100% of lifetime use in older adults and about 64% in the younger age groups (41). These differences in regulations and acceptance continue to shape social norms.

One limitation of our study was that we could not determine whether students' perceptions changed before the actual substance use behavior or whether perceptions changed as a result of the change or adjustment in behavior. Our sample was a convenience sample and only 2.4% of the enrolled students at Universities completed our baseline survey. Therefore, the observed substance use prevalences are not representative for University students in Germany. As is the case in many internet-based interventions, we had a substantial dropout rate, with only 28% completing both baseline and follow-up questionnaires. Although we were left with reasonable numbers for detailed statistical analyses, this is a potential source of bias which was previously reported for SN-studies (42). However, we were not able to detect small effects as intended. The substance use prevalence for most illicit substances (except for cannabis) assessed in our study was too low to run comparative analyses. Investigating the impact of SN interventions on illicit substances requires further research activities taking low prevalence rates into account. Also, external validity of our results is limited. However, our results add to the large body of evidence demonstrating misperceptions of peer substance use in representative samples and intervention effects of SN-approaches (43). Further, in our study, we did not treat intervention Universities any different from control Universities in terms of attempting to boost participation in the follow-up assessments. Another limitation was that the perceptions of personal and peer substance use, as well as the prevalence of substance use, were not assessed at the delayed intervention control Universities after students there had completed the web-based SNF. Therefore, we do not know whether students at these Universities experienced similar or different changes in the use of licit and illicit substances after 5 months as those noted for students at intervention Universities. Social contacts between students of intervention vs. control Universities in each region of Germany were possible, in principle, but were considered minimal by local study staff. Therefore, we do not think that cross-contamination of intervention effects occurred. Overall, differences in terms of courses offered and size of the student population between the included Universities have to be acknowledged, but we believe that these differences were of limited relevance to the grouped comparisons of intervention and control Universities.

The web-based PNF included feedback on both descriptive and injunctive norms. Any added effect of the injunctive norms feedback could not be determined in this study. Results of another study suggest that the combination of descriptive and injunctive norms feedback was as effective in reducing the frequency of drinking 2 weeks post-intervention as descriptive-norms-feedback only (20). Hence, a more parsimonious intervention only including the descriptive-norms feedback may suffice to achieve the desired effects on alcohol use in German University students. However, this needs to be the topic of further investigation in this population. Studies with factorial designs involving multiple cycles may be appropriate to test combinations of intervention components and to consecutively replace less effective or ineffective intervention components with effective ones (44).

To conclude, this study was the first cluster-controlled trial examining the impact of an evidence-based intervention addressing SN surrounding various substances targeting German University students. Findings of the INSIST-study suggest that a short web-based PNF can impact alcohol and cannabis use in this population. Contrary to a Cochrane Review on the effects of SN-interventions among University students which did not find meaningful benefits regarding alcohol misuse (45), our results provide a somewhat more positive picture, although effect sizes in our study were limited. Given the character of this low-threshold and comparatively easy to implement intervention at interested Universities, we recommend a more widespread implementation and detailed surveillance of intervention implementation and effects over longer periods of time in this setting.

## Data Availability Statement

The raw data supporting the conclusions of this article will be made available by the authors upon request.

## Ethics Statement

Because the study involved human participants, ethical approval was obtained from institutional review boards of all participating universities. Data protection was monitored by the local data protection agency in the city state of Bremen. Eight Universities in four regions participated in the study (Hamburg University of Applied Sciences, Hannover Medical School, University of Bielefeld, Heinrich Heine University Duesseldorf, Martin-Luther-University Halle-Wittenberg, Technical University Dresden, Heidelberg University, Mannheim University). The patients/participants provided their written informed consent to participate in this study.

## Author Contributions

CP: conzeptualization, investigation, writing—original draft preparation, validation, funding acquisition, and visualization. SH: conzeptualization, investigation, writing—original draft preparation, project administration, and visualization. SM: conzeptualization, investigation, writing—review, and editing. HZ: conzeptualization, investigation, writing—original draft preparation, supervision, and funding acquisition. HP: formal analysis, writing—original draft preparation, and visualization. SS: investigation, writing—review, and editing. RR, AS-P, MG, AK, AI, and UW: investigation, writing—review, and editing. All authors contributed to the article and approved the submitted version.

## Conflict of Interest

The authors declare that the research was conducted in the absence of any commercial or financial relationships that could be construed as a potential conflict of interest.

## Abbreviations

SN: social norms
PNF: personalized normative feedback.
